# Improving the Performance of Micro-Textured Cutting Tools in Dry Milling of Ti-6Al-4V Alloys

**DOI:** 10.3390/mi12080945

**Published:** 2021-08-11

**Authors:** Ze Wu, Youqiang Xing, Jiansong Chen

**Affiliations:** School of Mechanical Engineering, Southeast University, Nanjing 211189, China

**Keywords:** surface texturing, dry milling, Ti-6Al-4V alloys, power consumption

## Abstract

Micro-textured tools were fabricated by making textures on rake faces and filling them with molybdenum disulfide. Dry milling of Ti-6Al-4V alloys was carried out with the micro-textured tools and conventional tools for comparison. Results showed that micro-textured tools can reduce the resultant cutting forces, cutting temperatures, and power consumption by approximately 15%, 10%, and 5%, respectively. Meanwhile, the developed tools can improve tool lives by approximately 20–25%. The radial width of cut, the cutting speed, and the axial depth of cut all had statistical and physical effects on the energy consumption per unit of volume in dry milling of Ti-6Al-4V alloys, while the feed per tooth seemed to have no significant effect. The mechanism for improved performance of micro-textured tools can be mainly interpreted as their self-lubricating function.

## 1. Introduction

Dry cutting has obvious advantages in saving energy, protecting the environment, and protecting workers’ health [1,2]. However, because of no cooling effect of the cutting fluid, rapid tool wear often occurs in dry cutting operations [3]. Titanium alloy is a typical difficult-to-machine material because of its high specific strength and low thermal conductivity. In dry cutting of titanium alloy, cutting heat accumulates in the cutting area without being taken away and results in high cutting temperature. As a result, tool wear intensifies and tool life is reduced. Cemented carbide cutting tools have been proved to be suitable for cutting of titanium alloys. It is also thought that surface texturing on cemented carbide cutting tools can alleviate tool wear. There are many micro-pit arrays on the textured surface that can store lubricants and capture wear debris [4,5]. Surface texturing has also been proved to be effective in improving cutting performance.

Song et al. [6] made a micro-pool on the tool–chip contact area of cemented carbide tools using micro-electronic discharge machining and analyzed the influence of the micro-pool on the mechanical strength of cutting inserts by finite element analysis. Sugihara et al. [7] developed a milling cutter using femtosecond laser technology to form stripe grooves on the rake face, which showed high crater wear resistance during cutting of medium carbon steel. At the same time, their research also showed that there was a strong correlation between the wear resistance and the width of the unit of texture. Enomoto et al. [8] reported that anti-adhesion of aluminum alloy was obtained by a kind of nano-textured cutting insert in milling operations. Kümmel et al. [9] indicated that the built-up edge (BUE) of the tools can be stabilized in dry cutting of steel by fabricating a dimple array on the cutting insert, which is accompanied by slight wear compared to the untextured cutting insert. Xing et al. [10] fabricated a kind of surface-textured Al_2_O_3_/TiC ceramic turning insert that could reduce the cutting vibration and improve processing quality in cutting of hardened steel. Surface texturing can also exhibit superiority by being combined with coating technology. Viana et al. [11] fabricated micro-textures on the surface of cemented carbide inserts (ISO K grade) and then coated them with TiAlN and AlCrN. As a result, by using textured tools, the delamination of the coating was suppressed in face milling of compacted graphite cast iron, which resulted in prolonged tool life. Zhang et al. [12] also demonstrated the feasibility of fabricating micro-/nano-scale textures on tool substrate surfaces to improve the anti-adhesive wear properties of TiAlN-coated tools. Ma et al. [13] investigated the performance of microbump-textured cutting tools in dry turning of mild steel by AdvantEdge finite element simulation and indicated that the microbump width, microbump height, and edge distance all had an influence on the cutting force in their own ways.

The treatment of surface textures is an effective way to improve performance in cutting operations. However, the literature review shows that there is a lack of research on the cutting energy consumption when milling titanium alloy with surface-textured tools. In this study, a surface texture was prepared on cemented carbide inserts by high-energy beam machining to form so-called micro-textured tools. Dry milling of Ti-6Al-4V alloys was carried out, the used tools were so-called micro-textured and non-textured tools, and the results were compared. The machining performance was evaluated by combining cutting force, cutting temperature, surface roughness of the finished workpiece, tool life, and power consumption. The purpose of this study was to investigate the feasibility of surface texturing in improving the dry-milling performance of Ti-6Al-4V alloys, especially the influence of the so-called micro-textured tool on power consumption, which has gradually become the focus of machining operation.

## 2. Experimental

### 2.1. Preparation of Micro-Textured Cutting Tools

**Figure 1:**
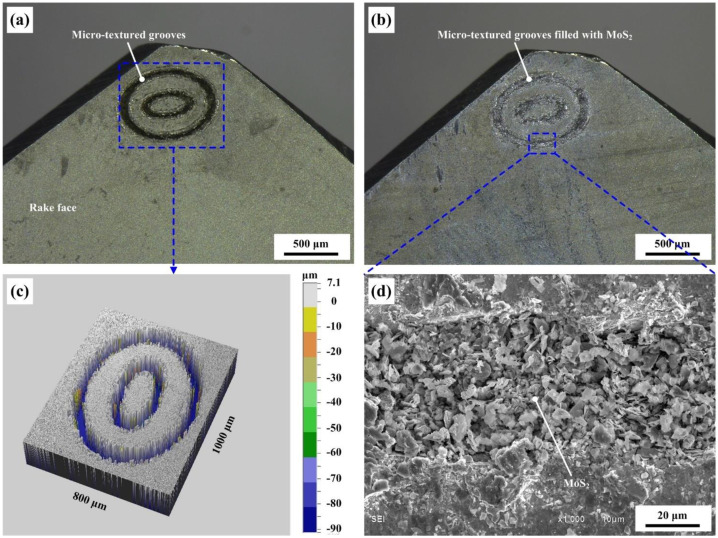
Micrographs of the micro-textured grooves: (**a**) without solid lubricant, (**b**) filled with molybdenum disulfide, (**c**) 3D topography, and (**d**) magnified topography of the grooves filled with molybdenum disulfide.

In the present study, the used cutting tool material was YG6 cemented carbide composition, and physical parameters of the selected cemented carbide are shown in Table 1. The rake angle, relief angle, clearance angle, tool tip radius, and cutting-edge radius of the cutting inserts were 0°, 20°, 5°, 0.5 mm, and 0.02 mm, respectively. The cemented carbide cutting inserts were first polished by an automatic polishing machine and then cleaned for 10 min on an ultrasonic cleaning machine. Surface textures were fabricated on the tool–chip contact area of the cutting inserts by a nano-second laser. The focal length of the nano-second laser was 65 mm, while the scan area was about 45 mm × 45 mm. The laser processing was carried out in the atmosphere, with a working voltage of 15 V, an average rated current of 20 A, and a scanning speed of 10 mm/s. The width of the single groove was about 50 μm, while the maximum depth was 100 μm. Molybdenum disulfide solid lubricant was filled into the grooves through special tools under microscopic observation. The micrograph of textured grooves without as well as filled with molybdenum disulfide are shown in Figure 1a,b, while the 3D topography of the grooves and the magnified topography of the grooves filled with molybdenum disulfide are given in Figure 1c,d, respectively. As shown in Figure 1, the textured pattern consisted of two ellipses. The long and short diameters of the big ellipse were 0.8 mm and 0.6 mm, respectively, while the long and short diameters of the small ellipse were 0.4 mm and 0.2 mm, respectively. The minimum distance between the edge of the big ellipse and the cutting edge of the tool was about 0.1 mm.

### 2.2. Milling Tests

Up-cutting tests were carried out on a vertical CNC machining center (DAEWOO ACE-V500). The rated power of the machining center was 15 kW, while the rated spindle speed was 10,000 rpm. Geometric parameters of the tool holder were composed of a radial rake angle of −5°, an axial rake angle of 20°, and a major cutting-edge angle of 45°. Only one tooth was used in one cutting operation. The used titanium alloys were annealed with a hardness of 30 HRC. The size of the workpiece was 200 mm × 150 mm × 50 mm, and one cutting process was completed along the side length of 200 mm in one test. First, single-factor tests were carried out to investigate the cutting performance of so-called micro-textured tools. In the single-factor tests, the cutting speed *v* was selected ranging from 200 to 360 m/min, while the radial width of cut *a*_e_, feed per tooth *f*_z_, and axial depth of cut *a*_p_ were fixed at 50 mm, 0.1 mm/z, and 1 mm, respectively. Furthermore, the Taguchi method was applied to investigate the influence of cutting parameters on power consumption. In the Taguchi tests, the cutting speed, radial width of cut, axial depth of cut, and feed per tooth were set in the range of 200–320 m/min, 15–80 mm, 0.5–2 mm, and 0.05–0.3 mm/z, respectively. The cutting forces were recorded by a KISTLER piezoelectric quartz dynamometer. The cutting temperatures were recorded by a TH5104R infrared thermal imager. The flank wear was measured by a 3D digital microscope (DINOLITE AT01-MSUSB401). The roughness of the machined surface was measured by a roughness-measuring instrument. A commercial power sensor was used to measure the power consumption in the milling process. The tests under each cutting condition were repeated three times.

## 3. Results and Discussion

### 3.1. Single-Factor Tests

#### 3.1.1. Resultant Cutting Force

**Figure 2:**
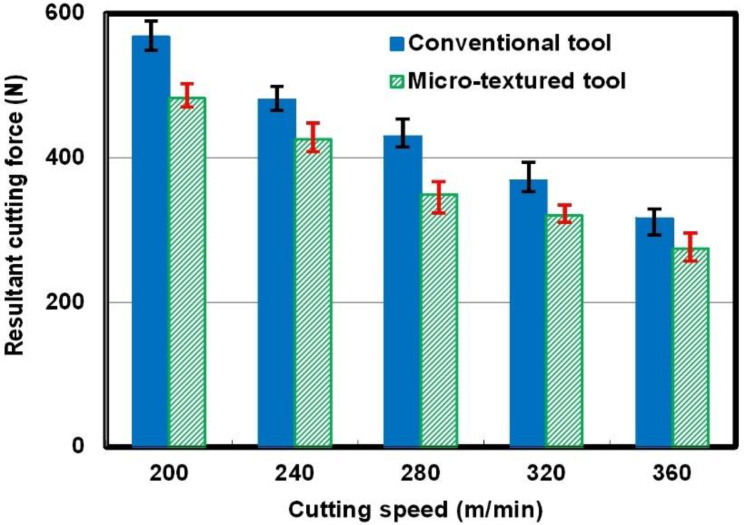
Resultant cutting forces of the micro-textured tool and the conventional tool at different cutting speeds.

The resultant cutting force was calculated based on three direction forces obtained in the milling process. Figure 2 illustrates the average values of the resultant cutting forces at different cutting speeds with the so-called micro-textured tool and the conventional one for comparison (*a*_e_ = 50 mm, *a*_p_ = 1 mm, and *f*_z_ = 0.1 mm/tooth). It can be found that the resultant cutting forces exhibit a decreasing tendency with an increase in cutting speed, which can be due to softening of the workpiece material at a higher cutting speed. It is obvious that the value of the cutting force of the micro-textured tool is smaller than the value of the conventional tool at the same cutting speed. The calculated results indicate that the average resultant cutting forces of the micro-textured tool are reduced by approximately 15% compared to those of the conventional tool. In other words, so-called micro-textured tools have the function of reducing cutting forces in dry milling of Ti-6Al-4V alloys.

#### 3.1.2. Cutting Temperature

**Figure 3:**
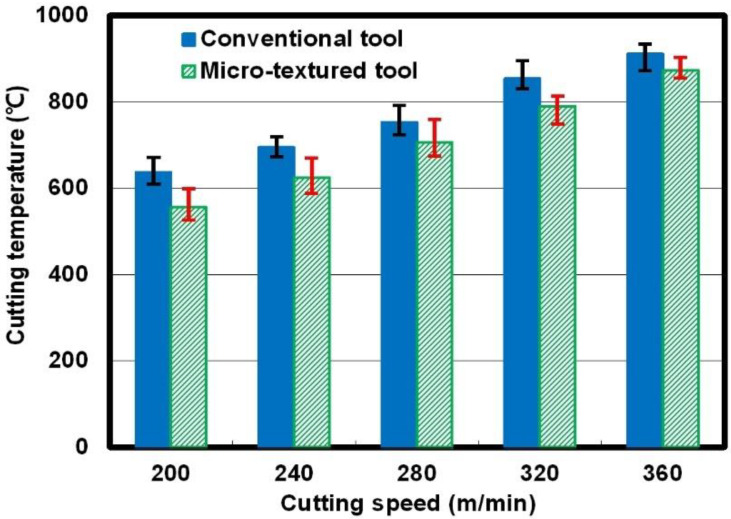
Cutting temperatures of the micro-textured tool and the conventional tool at different cutting speeds.

In the cutting process, the temperature distribution in the cutting area was measured at 5 s intervals by the TH5104R infrared thermal imager. The distance between the infrared thermal imager and the tool was 400 mm, and the infrared thermal imaging system was calibrated in advance. The mean value of the highest temperatures in each temperature field was treated as the cutting temperature. Figure 3 shows the cutting temperatures of the two kinds of cutting tools at different cutting speeds. It can be seen in Figure 3 that the cutting temperatures exhibited an increasing trend when the cutting speed increased. The value of temperature for the so-called micro-textured tool was lower than the value for the non-textured tool under the same cutting condition. For example, when the cutting speed was 200 m/min, the cutting temperature for the non-textured tool was 637.2 °C; however, the value was 556.1 °C for the micro-textured tool. The calculation presents that the cutting temperature can be reduced by 10% because of the surface texturing.

#### 3.1.3. Surface Roughness

**Figure 4:**
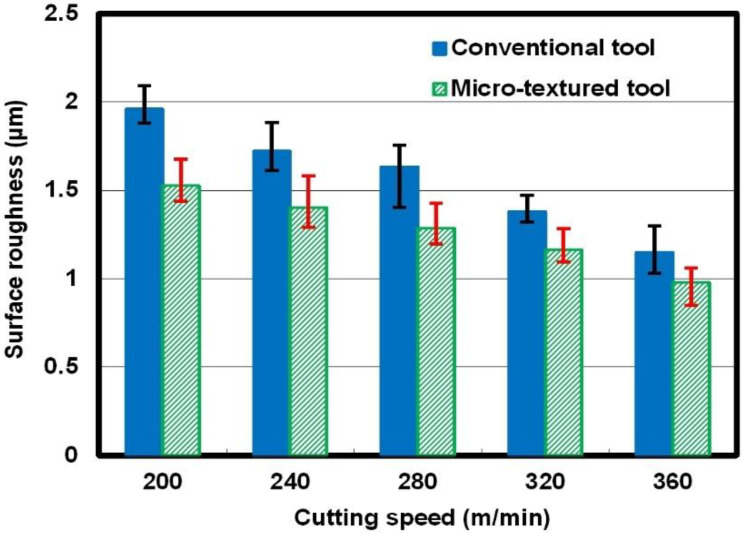
Surface roughness of the finished workpiece at different cutting speeds.

Roughness is a mean value of three measured points at different positions. Figure 4 illustrates the surface roughness measured at different cutting speeds. It is obvious from Figure 4 that surface roughness presented a decreasing tendency for both the micro-textured cutting tool and the non-textured one as the cutting speed increased from 200 m/min to 360 m/min. Under the same cutting condition, the value of the surface roughness of the finished workpiece for the micro-textured cutting tool was smaller than the value for the non-textured cutting tool. This means that the surface roughness of the finished workpiece can be reduced by using so-called micro-textured tools in dry milling of Ti-6Al-4V alloys.

#### 3.1.4. Tool Life

**Figure 5:**
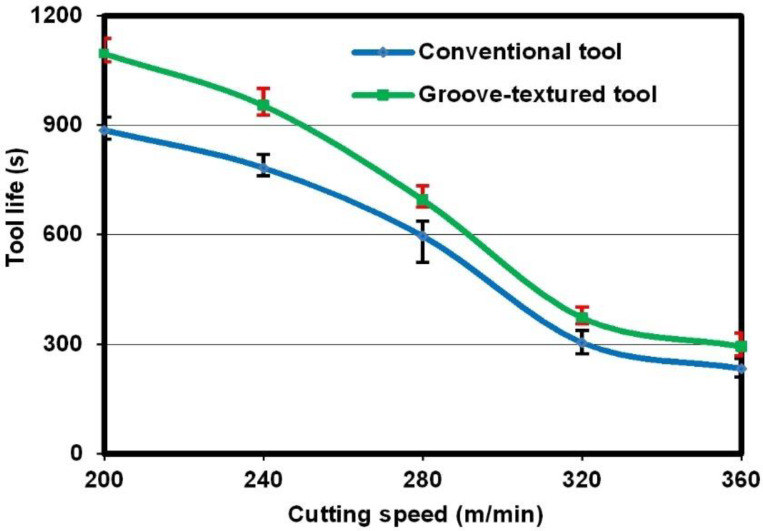
Tool lives obtained under different cutting conditions.

In the cutting of titanium alloys, flank wear as well as tip wear are the main failure modes for cemented carbide cutting tools. In the present study, the 600 μm maximum flank wear was selected as the criterion of tool failure according to the ISO 3685 standard [14]. If the flank wear of the cutting insert reaches 600 μm, the tool is considered invalid. Figure 5 shows the tool lives of two different kinds of cutting tools at different cutting speeds. It is obvious that the tool lives of all the tools decreased with the increase in cutting speed. However, compared to the non-textured tool, the micro-textured tool can prolong tool lives by approximately 20–25%.

#### 3.1.5. Power Consumption

**Figure 6:**
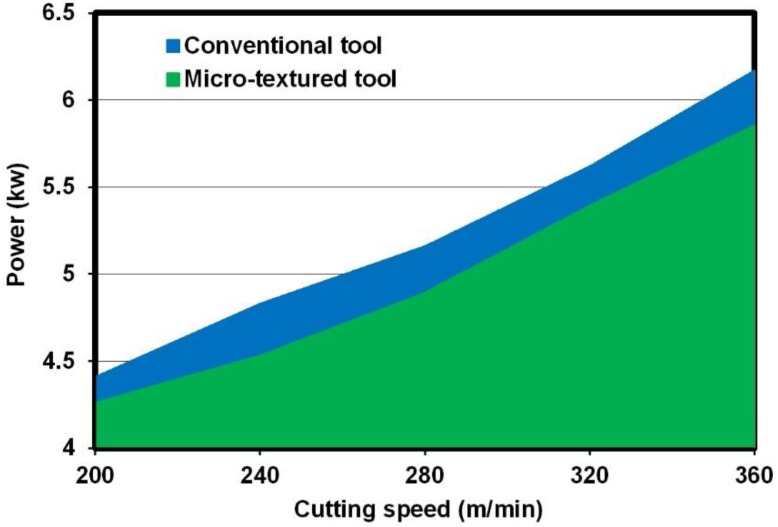
Comparison of power consumption of the used milling machine between operations with different cutting tools.

Figure 6 indicates the comparisons of power consumption of the used milling machine between operations with different cutting tools (*a*_e_ = 50 mm, *a*_p_ = 1 mm, and *f*_z_ = 0.1 mm/tooth). It can be seen from Figure 6 that the power consumptions of the used milling machine gradually increased when the cutting speed increased, either for the micro-textured tool or for the conventional one. For the milling operation with the conventional tool, the power consumption increased from 4.41 kW to 6.17 kW as the cutting speed increased from 200 m/min to 360 m/min. Moreover, at the same cutting speed, the power consumption for operation with the so called micro-textured tool reduced compared to the value for the non-textured tool. For example, at a cutting speed of 280 m/min, the power consumption for operation with the non-textured tool was 5.16 kW, while the value was 4.89 kW for operation with the micro-textured tool. The calculation shows that the using of micro-textured tools can reduce power consumption by approximately 5%. The reduction in power consumption may be due to the reduced cutting forces that are obtained by using micro-textured tools.

**Figure 7:**
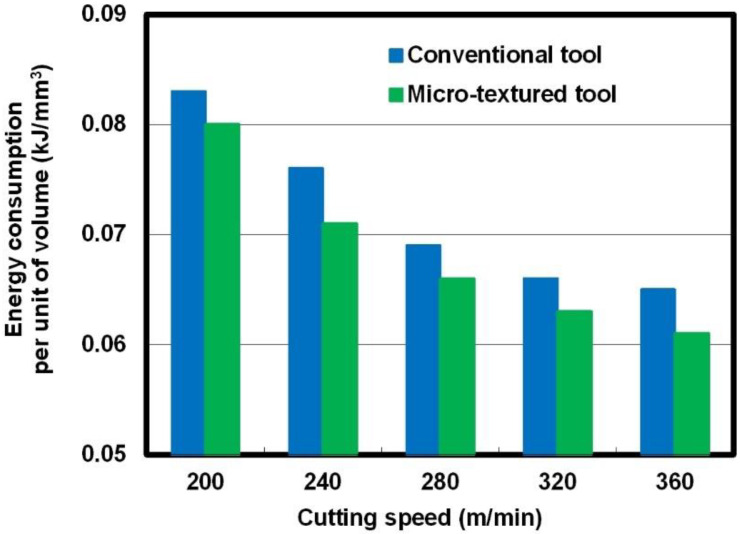
Energy consumption per unit of volume for operations with different cutting tools.

In the cutting process, the energy consumption per unit of volume can be expressed as power consumption divided by the material removal rate. Figure 7 illustrates the energy consumptions per unit of volume as a function of cutting speed with the so-called micro-textured tool and the conventional one for comparison. It is obvious that the energy consumption per unit of volume presented a decreasing tendency with an increase in cutting speed, which is opposite to the variation in power consumption. Moreover, at the same cutting speed, the energy consumption per unit of volume for operation with the micro-textured tool reduced compared to the value for the non-textured tool.

### 3.2. Taguchi Tests

The aforesaid experimental results indicate that the use of micro-textured tools can reduce the power consumption in the milling process. To further investigate the influence of cutting parameters on the energy consumption per unit of volume, an orthogonal experiment was conducted in the milling of Ti-6Al-4V alloys by so-called micro-textured tools. The experiment was conducted per the standard orthogonal array [15]. In the present investigation, an L_16_ orthogonal array that had 16 rows and 5 columns was chosen. The experiment consisted of 16 tests, and the columns were assigned to parameters. The first column was assigned to cutting speed, the second column was assigned to the feed per tooth, the third column was assigned to the axial depth of cut, the fourth column was assigned to the radial width of cut, and the last column was assigned to the test error. The experiment was conducted per the orthogonal array with the level of parameters. The test results were subject to analysis of variance.

The orthogonal array of parameters and the corresponding experimental results are shown in Table 2. Moreover, the results of analysis of variance for energy consumption per unit of volume are presented in Table 3. The seventh column of the analysis of variance for the energy consumption per unit of volume (see Table 3) indicates the percentage contribution (*P*) of each factor to the total variation, indicating its influence on the result. Table 3 shows the radial width of cut (*P* = 62.35%), the cutting speed (*P* = 29.96%), the axial depth of cut (*P* = 4.86%), the feed per tooth (*P* = 2.02%), and the error term (*P* = 0.81%).

According to Table 3, test *F* for the cutting speed (37) and test *F* for the radial width of cut (77) were all greater than the 99% confidence level (29.5); therefore, the cutting speed and the radial width of cut all produced a significant level within the reliability interval of 99%. In other words, the cutting speed and the radial width of cut have a significant effect on the energy consumption per unit of volume in the milling of Ti-6Al-4V alloys. The axial depth of cut had a certain effect on the energy consumption per unit of volume at the reliability interval of 90%. However, the feed per tooth had no effect on the energy consumption per unit of volume at the reliability interval of 90%. Conclusively, the radial width of cut, the cutting speed, and the axial depth of cut all have statistical and physical effects on the energy consumption per unit of volume in dry milling of Ti-6Al-4V alloys, while the feed per tooth seems to have no significant effect.

### 3.3. Discussion

In the present study, we developed so-called micro-textured tools and investigated their performance in dry milling of Ti-6Al-4V alloys. The results indicated that micro-textured tools are effective in reducing the cutting force, cutting temperature, power consumption, and surface roughness of the finished workpiece; meanwhile, operations with the developed tools also reduce tool wear and improved tool life. The action mechanisms of micro-textured tools are discussed below.

In the cutting process, friction forces between the chip and the rake face vary linearly with the tool–chip contact area and the average shear strength [16]. First, surface textures on the rake face of micro-textured tools can decrease the tool–chip contact area compared with conventional tools. Second, molybdenum disulfide may be released and smeared on the friction surface of micro-textured tools at high cutting temperature due to its high thermal expansion coefficient. The shear strength of molybdenum disulfide is lower than that of cemented carbide; as a result, friction forces can be reduced with the application of micro-textured tools. Reduced friction forces result in a reduction in the resultant cutting forces.

As mentioned above, tool–chip frictions can be reduced due to the lower shear strength of molybdenum disulfide. In other words, the wear resistance of micro-textured tools can be improved. As a result, the tool lives of micro-textured tools improve in dry milling of Ti-6Al-4V alloys. The lower surface roughness of the finished workpiece obtained by using micro-textured tools is also attributed to the lower tool wear.

It has been reported that shear deformations of chips can be reduced by the self-lubricating function of textured tools. In the metal-cutting process, the cutting heat is produced by shear deformations of the chips, tool–chip frictions, and tool–workpiece frictions. As a result, the cutting temperatures of micro-textured tools can be reduced due to the minor shear deformations of the chips as well as the reduced tool–chip frictions. Meanwhile, the heat-radiating areas are increased by fabrication of surface textures [4], which may reduce the cutting temperature by expediting the heat dissipation as a result.

It has been shown that the resultant cutting forces and the shear deformations of the chips can be reduced by using micro-textured tools. These may be the main reasons why power consumption is reduced in the milling process with micro-textured tools.

## 4. Conclusions

Surface textures were fabricated on the rake faces of cemented carbide inserts by laser beam machining, and the textures were filled with molybdenum disulfide to form so-called micro-textured tools. Dry milling of Ti-6Al-4V alloys was carried out with the micro-textured tools and conventional tools for comparison. From the experimental results and discussion, the following conclusions can be drawn:

1. Compared to conventional tools, micro-textured tools can reduce the resultant cutting forces and the cutting temperatures by 15% and 10%, respectively. The tool lives of so-called micro-textured tools are improved by approximately 20–25%. Meanwhile, the developed tools can also reduce the surface roughness of the finished workpiece to some extent.
2. The use of micro-textured tools can reduce the power consumption by approximately 5%. The cutting speed and the radial width of cut all have a certain effect on the energy consumption per unit of volume within the reliability interval of 99%. The axial depth of cut has a certain effect on the energy consumption per unit of volume at the reliability interval of 90%. However, the feed per tooth has no effect on the energy consumption per unit of volume at the reliability interval of 90%.
3. The mechanism for improved performance of micro-textured tools can be mainly interpreted as their self-lubricating function.

## Figures and Tables

**Table 1:** Composition and physical parameters of the selected cemented carbide.

Composition (wt%)	Hardness (HRA)	Thermal Conductivity (W/m·k)	Thermal Expansion Coefficient (10^−6^/k)	Density (g/cm^3^)
WC + 6%Co	92.0	52.3	4.8	15.4

**Table 2:** Orthogonal array of cutting parameters and corresponding experimental results of energy consumption per unit of volume.

Test	Cutting Speed, *v* (m/Min)	Feed per Tooth, *f*_z_ (mm/z)	Axial Depth of Cut, *a*_p_ (mm)	Radial Width of Cut, *a*_e_ (mm)	Error Term	Energy Consumption per Unit of Volume, *W* (kJ/mm^3^)
1	200	0.05	0.5	15	1	0.082
2	200	0.1	1	30	2	0.074
3	200	0.2	1.5	50	3	0.068
4	200	0.3	2	80	4	0.063
5	240	0.05	1	50	4	0.076
6	240	0.1	0.5	80	3	0.071
7	240	0.2	2	15	2	0.083
8	240	0.3	1.5	30	1	0.079
9	280	0.05	1.5	80	2	0.071
10	280	0.1	2	50	1	0.075
11	280	0.2	0.5	30	4	0.081
12	280	0.3	1	15	3	0.092
13	320	0.05	2	30	3	0.065
14	320	0.1	1.5	15	4	0.078
15	320	0.2	1	80	1	0.063
16	320	0.3	0.5	50	2	0.069

**Table 3:** Analysis of variance for energy consumption per unit of volume.

Source of Variance	Sum of Squares (×10^−6^)	Degree of Freedom	Variance (×10^−6^)	Test *F*	*F*	Percentage of Contribution (%)
Cutting speed	296	3	98.7	37	5.39 ^a^	29.96
Feed per tooth	20	3	6.7	2.5	9.28 ^b^	2.02
Axial depth of cut	48	3	16	6	29.5 ^c^	4.86
Radial width of cut	616	3	205.3	77		62.35
Error	8	3	2.7			0.81
Total	988	15				100

^a^ 90% confidence level. ^b^ 95% confidence level. ^c^ 99% confidence level.

## Data Availability

The data presented in this study are available on request from the corresponding author.
